# From virtual screening to animal models: chlorhexidine and indinavir as promising anti-Zika drug candidates

**DOI:** 10.3389/fcimb.2025.1699057

**Published:** 2026-01-30

**Authors:** Hai-Ting Zhang, Zhe-Yu Peng, Jun-Jun Xiong, Yang Luo, Jia-Hua Liu, Yi-Nan Du, Yin-Xu Hou, Sheng-Qun Deng

**Affiliations:** 1 Department of Pathology, The Second Affiliated Hospital of Anhui Medical University, Hefei, China; 2 Department of Pathogen Biology, Anhui Province Key Laboratory of Zoonoses, The Provincial Key Laboratory of Zoonoses of High Institutions in Anhui, School of Basic Medical Sciences, Anhui Medical University, Hefei, China; 3 Hunan Key Laboratory of Green Packaging and Application of Biological Nanotechnology, Hunan University of Technology, Zhuzhou, China; 4 Department of Disinfection and Vector Control, Anhui Provincial Center for Disease Control and Prevention, Hefei, China

**Keywords:** chlorhexidine, indinavir, molecular docking, NS2B/NS3 protease, virtual screening, Zika virus

## Abstract

**Introduction:**

Zika virus (ZIKV) infection is associated with severe neurological complications, but no clinically approved antiviral therapies exist, leaving management reliant on symptomatic support. The essential NS2B/NS3 protease represents a promising drug target for ZIKV.

**Methods:**

We performed structure-based virtual screening of 5,980 FDA-approved compounds from the ZINC database against the ZIKV NS2B/NS3 protease. Molecular docking identified 10 high-affinity candidates (LibDock score >150), which were subsequently evaluated for cytotoxicity and antiviral activity in Vero cells. The most promising compounds were further validated using immunofluorescence and Western blot assays. Their in vivo efficacy was assessed in a lethal AG6 mouse model.

**Results:**

Chlorhexidine and indinavir exhibited potent anti-ZIKV activity in vitro, with EC50 values of 16.41 µM and 12.8 µM, respectively, and favorable selectivity indices (CC50: 57.56 µM and 38.96 µM). Both compounds demonstrated a dose-dependent inhibition of ZIKV replication (5–40 µM) at the protein level. In the AG6 mouse model, treatment with either compound (50 mg/kg/day) significantly prolonged survival (p<0.001), delayed disease-associated weight loss, and reduced viral loads in key tissues compared to untreated controls.

**Discussion:**

Our integrated computational and experimental approach identifies chlorhexidine and indinavir as promising repurposed anti-ZIKV agents. While toxicity concerns require further investigation, these findings provide a validated foundation for the development of therapeutics against ZIKV infection.

## 1 Introduction

Zika virus (ZIKV) is a mosquito-borne flavivirus within the Flaviviridae family of positive-sense single-stranded RNA viruses (Miner and Diamond, 2017), primarily transmitted by *Aedes* mosquitoes (Musso and Gubler, 2016; Tajik et al., 2024). ZIKV was first observed in rhesus monkeys in Uganda in 1947 and was initially reported in people in 1952 (Lackritz et al., 2025; Wikan and Smith, 2016). ZIKV infection can cause many severe symptoms, including neurological implications, such as Guillain–Barré syndrome (GBS) in adults and neonatal microcephaly in newborns of infected pregnant women (Cao-Lormeau et al., 2016; Mehta et al., 2018). From 2013 to 2015, ZIKV triggered successive epidemics worldwide. An outbreak in French Polynesia affected approximately 28,000 individuals (Cao-Lormeau et al., 2014), followed by a larger-scale epidemic in Brazil with an estimated 440,000 to 1.3 million infections (de Araújo et al., 2016). Although global Zika virus disease cases have shown an overall decline since 2017, localized outbreaks were again reported in 2024 in countries including Thailand and India, indicating the persistent transmission of the epidemic (de Jong and Grobusch, 2025; Deshpande et al., 2025). Currently, there are no approved vaccines or specific therapeutic treatments for the Zika virus available for clinical use (Qin et al., 2024). The emergence of ZIKV and its large-scale transmission have posed a significant threat to public health (Roiz et al., 2024). Therefore, there is a pressing need to develop safe and effective therapeutic strategies against ZIKV. Therefore, there is a pressing need to develop safe and effective therapeutic strategies against ZIKV.

ZIKV is a single-stranded RNA virus with a genome size of approximately 10.8 kb and a length of approximately 3,423 amino acids. The viral RNA encodes a polypeptide precursor that can be cleaved by proteases, and its amino-terminal 1/3 produces three structural proteins, which are present in the capsid (C), pre-membrane (prM), and envelope (E) of the virion. The carboxy-terminal 2/3 of the polyprotein produces seven non-structural proteins, which are NS1, NS2A, NS2B, NS3, NS4A, NS4B, and NS5 (Michita et al., 2025; Sirohi and Kuhn, 2017). Among these proteins, NS2B and NS3 are involved in viral replication and maintenance of protein function (Santos et al., 2023). Furthermore, the NS3 protein is the second largest protein, which includes a helicase domain at the C-terminus and a protease domain at the N-terminus (Starvaggi et al., 2024). Part of the hydrophilic core region of the NS2B protein binds to the N-terminus of NS3 to form an NS2B/NS3 protease complex and works together. The NS2B-NS3 protease catalyzes the majority of cytoplasmic cleavages during ZIKV polyprotein processing, including the junctions NS2A-NS2B, NS2B-NS3, NS3-NS4A, and NS4B-NS5, as well as intramolecular sites within the capsid, NS2A, and NS4A proteins (Phoo et al., 2016). The NS2B-NS3 protease complex fulfills an essential function in viral replication: It mediates cleavage of the polyprotein precursor (PP) during the viral life cycle and enhances viral replication by facilitating the cleavage and degradation of host proteins (Sun et al., 2025). The NS2B/NS3 protease catalyzes the processing of polypeptides by viral precursors and plays a key role in the entire life of the ZIKV, making it an ideal drug target (Coluccia et al., 2020; Kumar et al., 2018; Shiryaev et al., 2023). While protease-targeting inhibitors are clinically approved for infectious diseases such as human immunodeficiency virus (HIV) and hepatitis C virus (HCV), and preclinical drug repurposing efforts have identified potential anti-ZIKV agents, no targeted therapy has yet been approved for Zika virus infection (Lin et al., 2023).

Currently, with the development of science and technology, computer-aided drug design (CADD) is being rapidly developed and is being gradually applied in various fields of drug research and development. Virtual screening technology has gradually become mainstream and is supported by a large amount of experimental data, which guarantees the efficiency and safety of the compounds to the greatest extent. Extensive virtual screening integrated with biological validation assays has led to the identification of numerous putative anti-ZIKV therapeutics (Chen et al., 2023; Santos et al., 2020; Singh and Jana, 2017). Molecular docking technology, by elucidating the interactions between receptors and ligands and their spatial binding conformations, has evolved into a core methodology in the field of drug discovery and screening (Li et al., 2022; Pinzi and Rastelli, 2019). Crystalline complexes of the NS2B/NS3 protease and borate inhibitor provide the active site and platform for screening antiviral compounds (Lei et al., 2016). Thus, we performed structure-based virtual screening targeting the binding site of the NS2B/NS3 protease. According to the docking scores and stability between protein targets and molecular ligands, 10 chemical compounds from the ZINC were screened. Based on the combined assessment of cytotoxicity and antiviral activity, we demonstrated that both chlorhexidine and indinavir effectively inhibit ZIKV replication within selected concentration ranges. Furthermore, *in vivo* evaluations conducted in an established ZIKV-infected mouse model substantiated that both compounds exhibit antiviral efficacy, indicating their potential for development into more effective anti-ZIKV therapeutic agents.

## 2 Materials and methods

### 2.1 Cells, viruses, and compounds

Vero cells (African Green Monkey kidney) were recovered and cultured in Dulbecco’s modified Eagle medium (DMEM) (Gibco, USA) supplemented with 10% fetal bovine serum (FBS) (Beyotime Biotechnology, China), 100 IU penicillin, and 0.1 mg/mL streptomycin at 37°C in a 5% CO_2_ humidified environment. The Asian lineage of ZIKV (strain Z16006; GenBank no. KU955589.1) was a generous gift from Dr. Changwen Ke from the Institute of Microbiology, Centers for Disease Control and Prevention of Guangdong Province, China. ZIKV was grown at 28°C with three rounds of amplification in C6/36 cells, with each round lasting 7 days. The cells were lysed and harvested, and the supernatant was stored at −80°C. A total of 10 compounds (Topscience Biotechnology, China) selected from the top-scoring list were obtained from the ZINC compound library and dissolved in DMSO (New Cell & Molecular Biotechnology, China) and stored at 4°C.

### 2.2 Molecular docking

The Food and Drug Administration (FDA)-approved molecular ligands were downloaded from the ZINC database (http://zinc15.docking.org/), and the NS2B/NS3 protease (PDB ID 5LC0) was downloaded from the PDB database (https://www.rcsb.org/) (**Supplementary Figure 1**). After importing molecule ligands into the DS software, water molecules, the same subunits, and small molecule ligands in the symmetrical double-stranded structure of the target protein are deleted, and only one chain and its small molecule ligands are retained. After removing the endogenous small-molecule ligand (6T8), molecular docking between the prepared small-molecule ligands and active sites was performed. In this experiment, the maximum number of hits was set to 50, and the other parameters were set to the default values. According to the docking score and the stability between the target and molecular ligands, 10 compounds were selected for this experiment.

### 2.3 Cytotoxicity and antiviral effects of the compounds

To assess the cytotoxicity of compounds, Vero cells were cultured in a 96-well plate and treated with different concentrations of compounds for 48 h, 10% CCK-8 (Gooniebio, China) reagent was added and incubated in a CO_2_ incubator for 1.5 h, and the absorbance was measured at 450 nm by CMax Plus (Molecular Devices, USA). The CC_50_ was calculated as the concentration required to reduce cell viability by 50%. For the antiviral effect of compounds, Vero cells were grown at 1× 10^4^ cells/well in a 96-well culture for 24 h, and each well was infected with ZIKV at an MOI of 5 for 2 h. Then, different concentrations of compounds were added to each well, and the cells were incubated for 48 h. All compounds were dissolved in DMSO and subjected to serial dilution in cell-based assays, ensuring that the final DMSO concentration in all treatment wells did not exceed 0.5% (v/v). At this concentration, DMSO exhibited no significant impact on Vero cell viability or viral replication. The cell supernatant was harvested 48 h postinfection, and total cellular RNA was extracted by the EasyPure^®^ Viral DNA/RNA Kit (TransGen Biotech, China) according to the manufacturer’s instructions. EC_50_ was calculated as the compound concentration required to reduce viral yields by 50%.

### 2.4 Immunofluorescence microscopy

Vero cells were grown at 2×10^4^ cells/well in a 24-well culture plate, and each well was infected with ZIKV at an MOI of 5 for 2 h. Then, different concentrations of compounds were added to each well, and the cells were incubated with Vero cells for 48 h. Vero cells were washed in PBS three times and fixed with 4% paraformaldehyde for 30 min at room temperature. The cells were then blocked with 5% bovine serum albumin (BSA) (Beyotime, China) for 30 min at 37°C. The cells were washed three times and incubated with an anti-flavivirus E-glycoprotein antibody (Abcam, USA) diluted 1:200 in PBS at 4°C for 18 h. Then, the 24-well culture plate was placed in an incubator at 37°C for 30 min, after which the cells were washed with PBS three times. The cells were then incubated with goat anti-mouse IgG (Abways Technology, China) for 2 h, after which the cells were washed three times with PBS. The nuclear staining dye DAPI (Beyotime, China) was added, and the sections were incubated for 5 min. Fluorescence images were recorded under a fluorescence microscope with an Olympus DP73 imaging system (Olympus, Japan).

### 2.5 Animals experiments

4–6-week-old I/II-type interferon receptor-deficient (Ifnagr^−^/^−^) C57BL/6 (AG6) (GemPharmatech, China) mice were selected for the experiment. Mice were randomly divided into three groups (n=5/group): the indinavir treatment group, the chlorhexidine treatment group, and the untreated control group. All mice were inoculated with ZIKV via intraperitoneal injection at a dose of 1×10^5^ PFU. Starting 24 h postinfection, mice in the treatment groups received daily drug administration by gavage (indinavir or chlorhexidine, both at 50 mg/kg). Untreated control group mice were administered an equal volume of phosphate-buffered saline (PBS) via gavage during the same period. Body weight changes and survival status were monitored daily postinfection. At the experimental endpoint, all mice were subjected to humane euthanasia by cervical dislocation immediately following deep anesthesia induced by intraperitoneal injection of 1% pentobarbital sodium solution (50 mg/kg). Upon euthanasia, tissue samples from the heart, liver, spleen, lungs, and kidneys were promptly harvested, rapidly frozen in liquid nitrogen, stored at −80°C, and subsequently analyzed by quantitative PCR (qPCR) for viral load determination. All animal procedures were approved by the Animal Ethics Committee of Anhui Medical University (approved code: LLSC20241679).

### 2.6 Statistical analysis

All the data are presented as the means ± SDs and were analyzed by two-way ANOVA, followed by the Tukey test, where a *p-value* < 0.05 represented a significant difference. These data are representative of three to five independent assays performed in duplicate. All analyses were performed with GraphPad Prism 8.0 software (GraphPad Software Inc., San Diego, CA, USA).

## 3 Results

### 3.1 Molecular docking results

The binding conformations of compounds to the target protein were scored using the LibDock module in Discovery Studio software. The LibDockScore is calculated based on polar (hydrogen bonding) and non−polar (hydrophobic) features of protein–ligand interactions, with higher scores indicating stronger predicted binding affinity. In this screening, we set a score >150 as the initial threshold to select the top-ranked molecules from 5,980 compounds for subsequent analysis. This threshold aims to prioritize candidate compounds with potential high affinity from a large number of compounds (**Table 1**).

**Table 1:** The relative information of the 10 compounds tested.

Molecular name	Molecular weight (g/mol)	Absolute energy (kcal/mol)	Relative energy (kcal/mol)	LibDock score	Molecular structure (2D)
Cobicistat	776.0	57.7454	5.38411	178.166	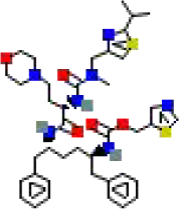
Indinavir	613.8	85.9878	4.08775	169.232	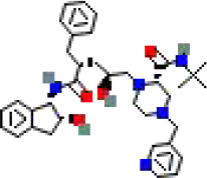
Deferoxamine	560.7	25.8121	8.51685	166.754	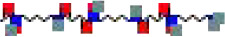
Carfilzomib	719.9	68.1915	5.80782	166.09	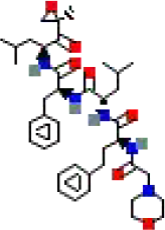
Chlorhexidine	505.4	56.7561	7.73922	161.574	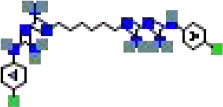
Atazanavir	704.9	121.503	7.59296	161.222	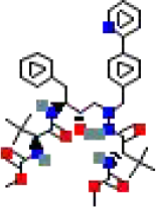
Valrubicin	723.6	89.8246	6.80535	157.728	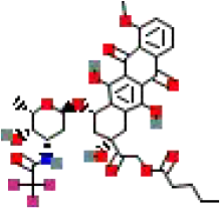
Mitoxantrone	444.5	77.2251	13.8628	156.613	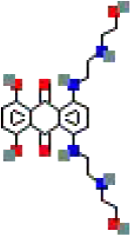
Naloxegol	651.8	85.3146	12.5686	154.014	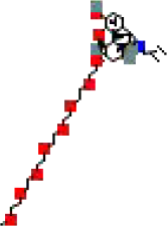
Folotyn	477.6	67.1417	12.8261	150.958	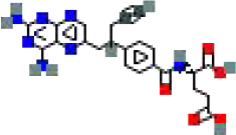

### 3.2 Cytotoxicity and antiviral effects of the compounds

**Figure 1:**
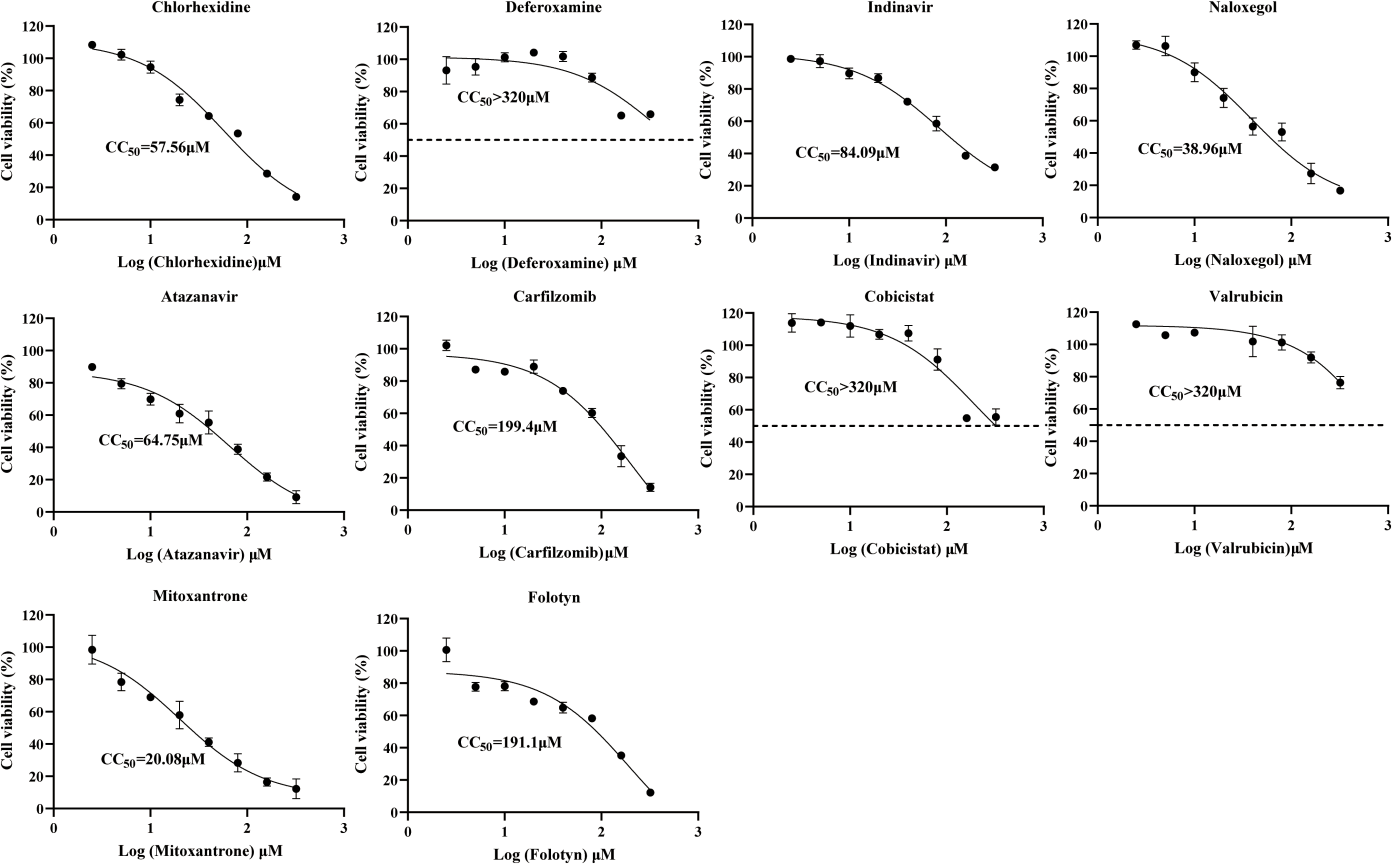
Cytotoxicity evaluation of 10 compounds. The cell viability of Vero cells was treated with different concentrations of 10 compounds. The CC_50_ of the compounds was calculated by fitting the curve by GraphPad Prism 8.

The cytotoxic effects of the compounds on Vero cells were assessed using the CCK-8 assay. The results showed that within the concentration range of 5-320 μM, the cell survival rates of cobicistat, deferoxamine, and valrubicin were all above 50%, and their CC_50_ values were all greater than 320 μM. In contrast, the cell viability of indinavir, carfilzomib, chlorhexidine, atazanavir, mitoxantrone, naloxone, and folotyn decreased in a concentration-dependent manner. The CC_50_ of these compounds were respectively 84.09, 199.4, 57.56, 64.75, 20.08, 38.96, and 191.1 μM (**Figure 1**).

**Figure 2:**
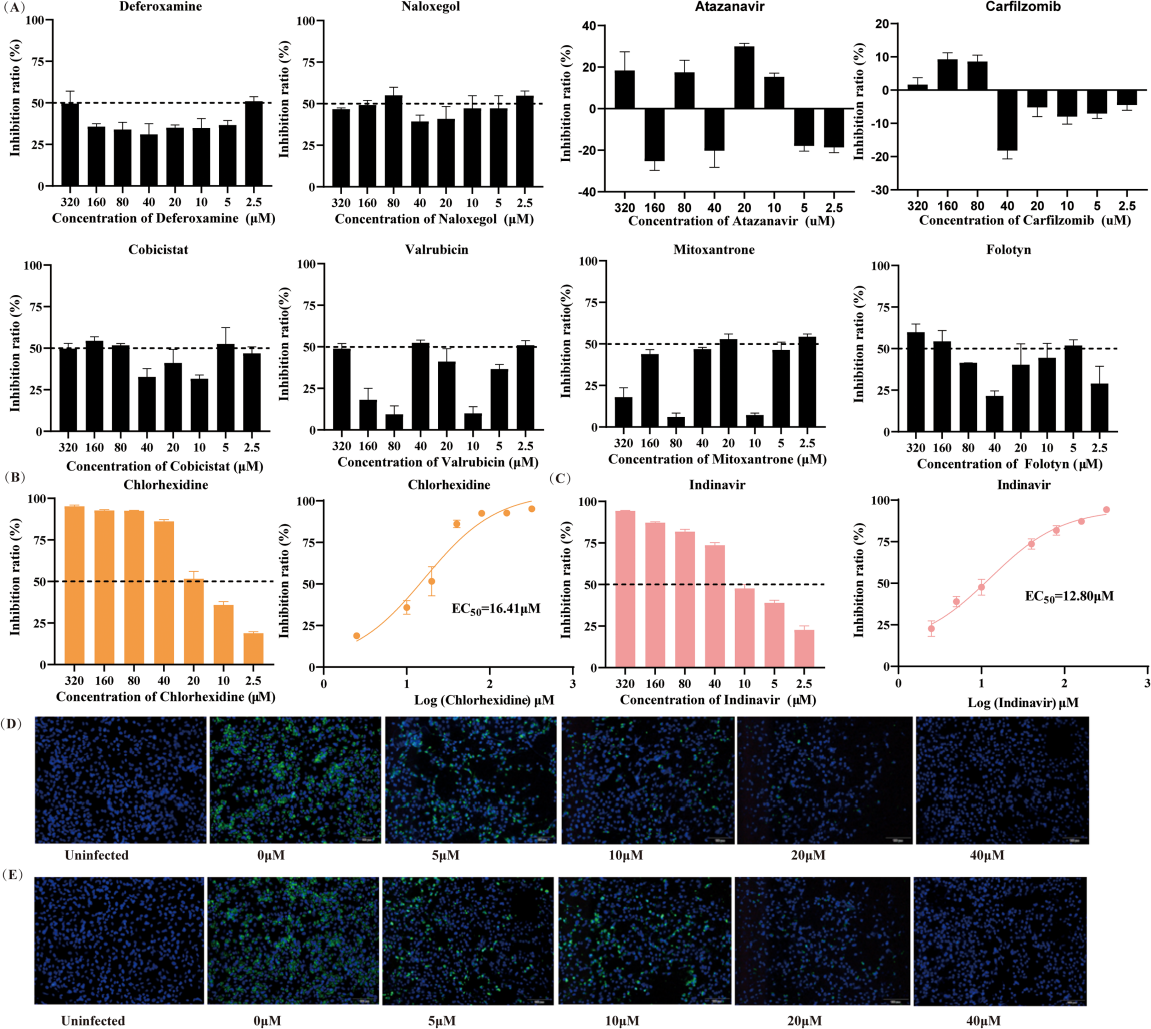
Assessment of compound antiviral activity. **(A-C)** Inhibition rates of different compound concentrations against ZIKV, compound CC50 values were calculated through curve fitting using GraphPad Prism 8. **(D, E)** Antiviral effects of indinavir and chlorhexidine against ZIKV were observed under immunofluorescence microscopy at concentrations ranging from 5 to 40 μM.

Although deferoxamine, naloxegol, cobicistat, valrubicin, mitoxantrone, and folotyn exhibited weak inhibitory effects against ZIKV at specific concentrations, the inhibition rates of these compounds across the concentration range of 2.5 to 320 μM showed no apparent concentration-dependent trend and were below 50%, indicating that they lack inhibitory effects against ZIKV infection (**Figure 2A**). Furthermore, no inhibitory activity was detected for atazanavir and carfilzomib. Chlorhexidine and indinavir both inhibited ZIKV within the concentration range of 2.5 to 320 μM, and the inhibitory effect diminished with decreasing concentration. The EC_50_ values for the two compounds were 12.8 μM (**Figure 2B**) and 16.41 μM (**Figure 2C**), respectively. The selection index (SI = CC50/EC50) serves as a preliminary indicator for evaluating the safety window of a compound. Although the SI > 1 suggests a certain therapeutic window within the tested concentration range, it is generally considered that compounds with the SI > 10 possess more favorable prospects for further development (Ojha et al., 2021). The SI of chlorhexidine and indinavir IS 3.51 and 6.57, respectively, indicating a relatively limited therapeutic window. This suggests that as lead compounds, their structures require further optimization to enhance selectivity and reduce cytotoxicity.

In subsequent immunofluorescence analyses, both chlorhexidine (**Figure 2D**) and indinavir (**Figure 2E**) exhibited inhibitory effects against ZIKV infection within the concentration range of 5-40 μM, with fluorescence intensity decreasing in a dose-dependent manner as the concentration increased.

### 3.3 The interaction map between proteins and compounds

**Figure 3:**
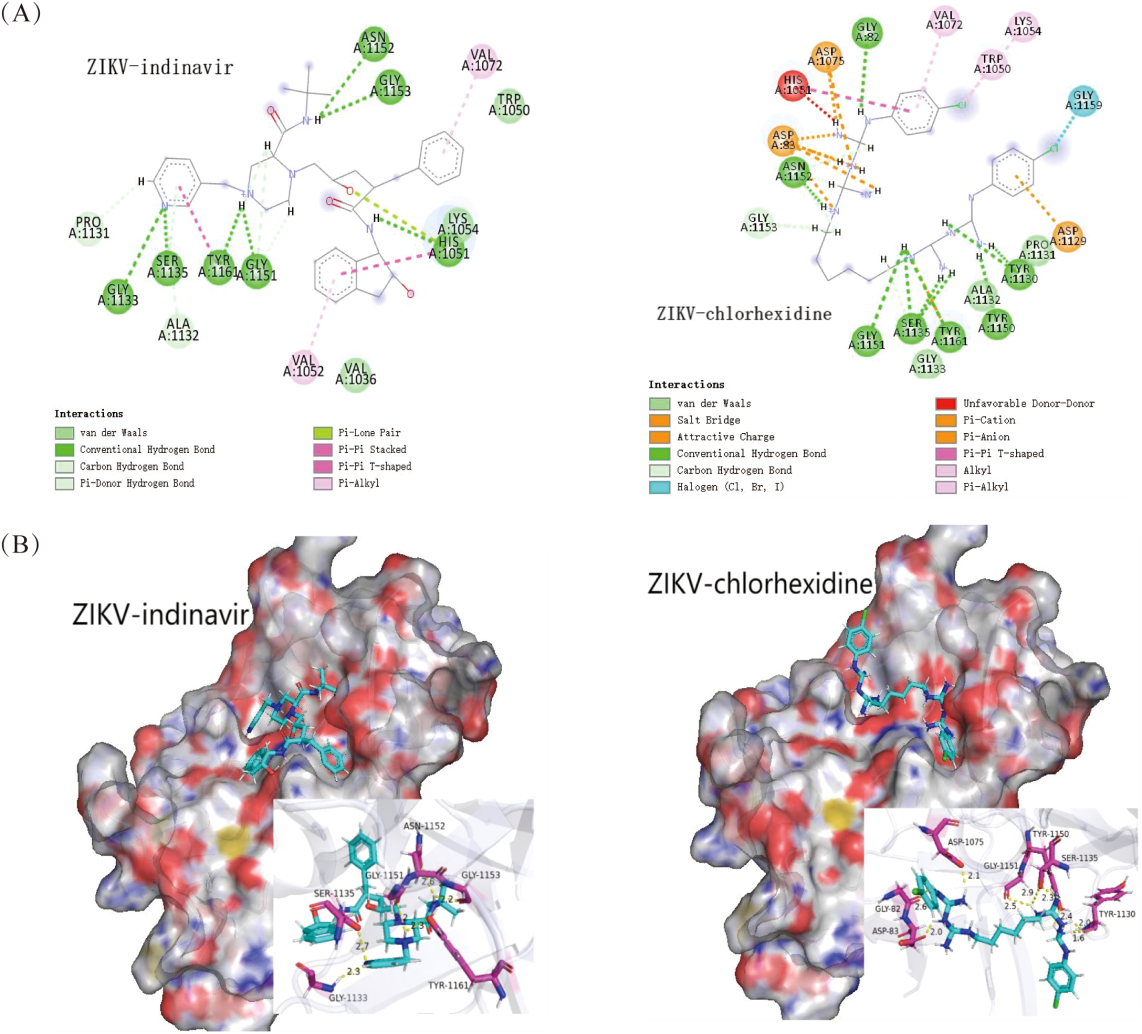
Molecular docking results of chlorhexidine and indinavir. **(A)** The 2D diagrams of indinavir and chlorhexidine were plotted by DS software. ASN, asparagine; GLY, glycine; Ser, serine; Tyr, tyrosine; His, histidine; Val, valine; Trp, tryptophan; Lys, lysine; Ala, alanine; Pro, proline. **(B)** The 3D interaction diagrams between target proteins and small molecules by PyMOL software.

PyMOL software was used to construct 3D plots of the interactions between target proteins and small molecules, and the hydrogen bond interactions and labels were used to display the hydrogen bond length and amino acid residues (**Figure 3A**). The interaction forces between the compounds and the target protein are shown in a 3D plot (**Figure 3B**), where the force types and hydrogen bonds could influence the bonding between the ligand and the receptor. Molecular docking studies demonstrate that both chlorhexidine and indinavir form stable binding conformations with the Zika virus (ZIKV) receptor protein. Chlorhexidine primarily interacts through hydrogen bonds with residues Asn 1152, Gly 82, Gly 1151, Ser 1135, Tyr 1161, Tyr 1150, and Tyr 1130; through hydrophobic interactions with residues His 1051, Val 1072, Trp 1050, and Lys 1064; and through van der Waals interactions with residues Gly 1133, Ala 1132, and Pro 1131 and forms a halogen bond involving its chlorine atom with residue Gly 1159. Indinavir primarily interacts through hydrogen bonds with residues Gly 1131, Ser 1135, Tyr 1161, Gly 1151, His 1051, Asn 1152, and Gly 1153; through hydrophobic interactions with residues Val 1072 and Val 1052; and through van der Waals interactions with residues Pro 1131, Ala 1132, Val 1036, Trp 1050, and Lys 1054. The molecular docking results of the remaining eight compounds are shown in **Supplementary Figure 2**.

### 3.4 Mice infected with ZIKV

**Figure 4:**
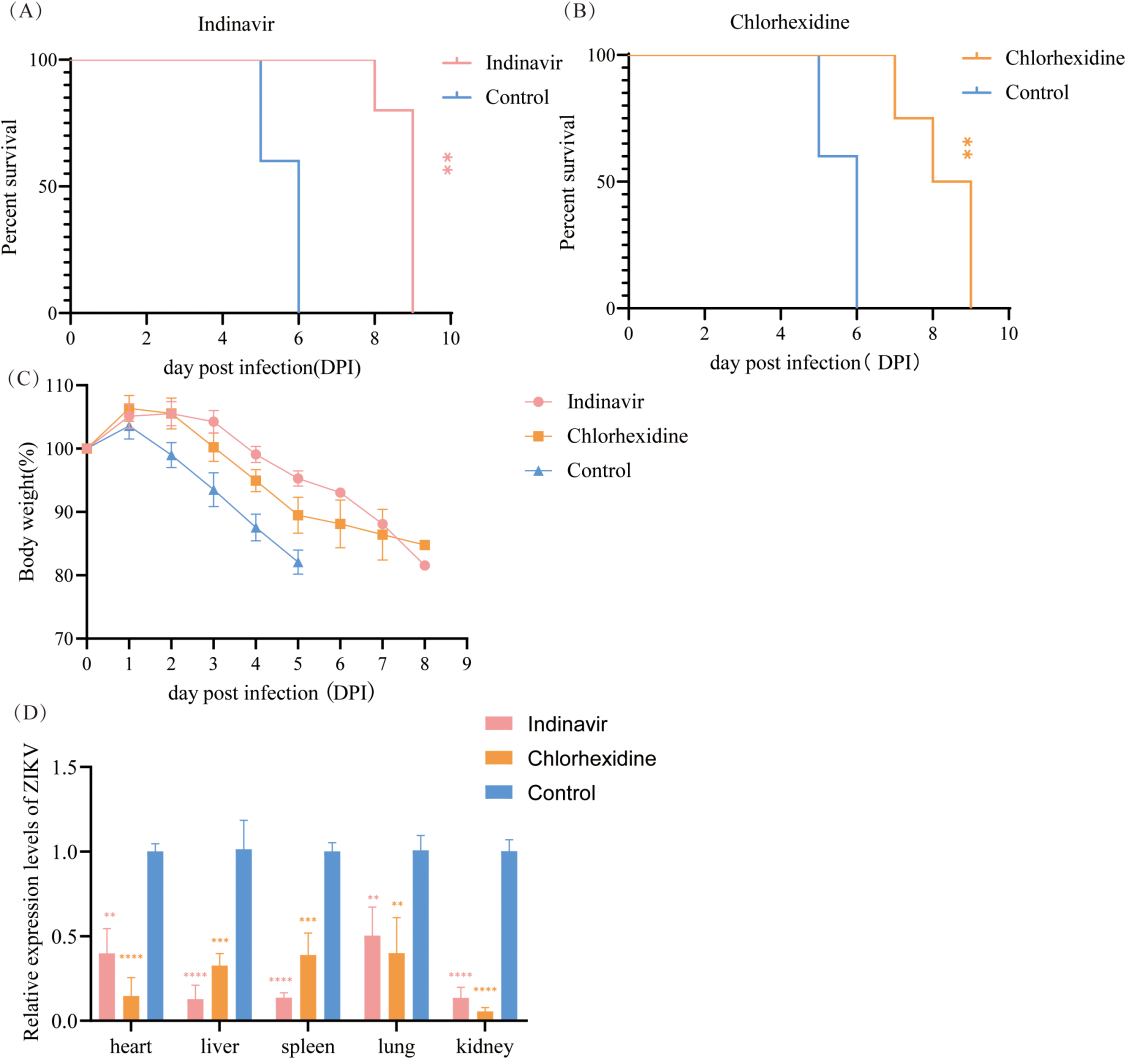
Efficacy of indinavir and chlorhexidine against Zika virus in mice. **(A)** Survival rate of ZIKV-infected mice treated with indinavir or PBS. **(B)** Kaplan–Meier survival rate of ZIKV-infected mice treated with chlorhexidine or PBS. **(C)** Body weight changes of ZIKV-infected mice treated with indinavir, chlorhexidine, or control vehicle. **(D)** Viral loads in various tissues of ZIKV-infected mice with or without treatment measured by qPCR. Statistical data were analyzed by two-way ANOVA, and a p-value ≤ 0.05 was considered to indicate statistical significance (*p < 0.05, **p < 0.01, ***p < 0.001,****p < 0.0001).

To evaluate the *in vivo* antiviral activity of indinavir and chlorhexidine, Zika virus-infected mice were administered the respective drugs at a dose of 50 mg/kg daily via oral gavage starting from 24 h postinfection. During the infection period, changes in body weight, clinical symptoms, and survival rates of the mice were monitored daily. All untreated control mice died within 6 days postinfection (**Figures 4A, B**). In contrast, treatment with either indinavir or chlorhexidine significantly prolonged the survival time of infected mice, with survival extended to day 9 postinfection (**Figures 4A, B**). Control mice exhibited significant weight loss starting on day 2 postinfection. However, mice in the drug-treated groups showed a delayed onset of weight loss until days 3-4 postinfection, and the rate of weight loss was slower (**Figure 4C**). At the experimental endpoint, all mice were euthanized, and tissue samples were collected. ZIKV RNA levels in tissues were detected by qPCR. Compared with the untreated control group, indinavir treatment significantly reduced ZIKV RNA loads across all examined murine tissues, with inhibition rates of 60.22% in the heart, 88.63% in the liver, 86.49% in the spleen, 50.41% in lung tissue, and 86.72% in the kidneys.

Similarly, chlorhexidine treatment induced a significant reduction in viral load, exhibiting inhibition rates of 85.45% in the heart, 68.76% in the liver, 61.29% in the spleen, 60.72% in lung tissue, and 94.68% in the kidneys (**Figure 4D**).

## 4 Discussion

Although ZIKV infection can cause severe neurological symptoms, there are currently no specific antiviral drugs or vaccines clinically available for its treatment. Virtual screening is widely applied in drug development. Its core principles involve elucidating the biological function of receptor targets and the mechanism of action of ligand compounds, thereby enabling the screening and identification of potential drug candidates based on known target structures or active ligand molecules (Macalino et al., 2015). Molecular docking is one of the most widely used methods in virtual screening, owing to its ability to predict the spatial conformation of small-molecule ligands within target binding sites with high accuracy (Meng et al., 2011). The ZIKV NS2B/NS3 protease plays a critical role in viral replication and represents a promising molecular target for anti-Zika virus drug development (Bernatchez et al., 2020; Nunes et al., 2022). Based on the high-affinity binding site characteristics of the ZIKV NS2B/NS3 protease, our study established a computer-aided screening approach to efficiently identify 10 potential anti-ZIKV candidate compounds from a library of 5,980 FDA-approved compounds. Subsequent evaluation of cytotoxicity and antiviral activity revealed that indinavir and chlorhexidine exhibited early-stage leads for anti-ZIKV candidate compounds.

In this study, we found that indinavir and chlorhexidine are effective antiviral compounds *in vitro*. It is worth noting that although treatment with indinavir and chlorhexidine significantly delayed disease progression and extended the median survival of infected mice from 6 to 9 days, all treatment group mice ultimately did not survive. This result may be related to multiple factors, including the following: (a) the selected dose did not reach or maintain a sufficient exposure level in the target tissue to completely inhibit virus replication; (b) the antiviral efficacy of compounds *in vivo* may be lower than *in vitro*; (c) the pathogenic mechanism of ZIKV infection is complex, and inhibition of a single target may not be sufficient to cope with late-stage infection. Future research could explore combination therapies with antiviral agents that have different mechanisms of action (e.g., viral entry inhibitors, RNA polymerase inhibitors) or conduct structural modifications of the lead compounds to improve their pharmacokinetic properties, potency, and selectivity. This study did not perform pharmacokinetic (PK) analysis; therefore, the systemic drug exposure level at this dose remains undetermined. Future studies should incorporate integrated PK/PD analyses to determine a more optimized dosing regimen. Also, the *in vitro* antiviral activity assessment in this study primarily relied on qPCR detection of viral RNA load. Future studies should further confirm the inhibitory effect of compounds on infectious viral particle production through methods such as plaque reduction assays.

Indinavir is a protease inhibitor drug clinically used for treating human immunodeficiency virus-infected patients (Plosker and Noble, 1999). It achieves antiviral activity through binding to the protease active site and inhibiting posttranslational processing, which results in the formation of immature non-infectious viral particles and interruption of viral spread (Boyd, 2007). Current evidence demonstrates that indinavir possesses potential therapeutic value for treating HPV-18-driven cervical carcinoma (Sharma et al., 2016). Furthermore, indinavir demonstrates favorable binding affinity to SARS-CoV-2 RNA-dependent RNA polymerase (RdRp), indicating its potential as a treatment for COVID-19 (Indu et al., 2020). Chlorhexidine is a bisbiguanide antimicrobial agent extensively applied in clinical disinfection. Additionally, chlorhexidine serves as an important adjunctive agent in periodontal disease treatment (Abbood et al., 2023; Poppolo Deus and Ouanounou, 2022). The mechanism of action of chlorhexidine is mainly because it can alter cell membrane permeability, ultimately inactivating viruses (Brookes et al., 2020).

While the compounds chlorhexidine and indinavir are relatively effective and safe when combined with the CC_50_ and EC_50_ values in this study, the potentially severe effects they cause are still worth considering. When considering reusing chlorhexidine and indinavir for anti-ZIKV treatment, their known potential adverse reactions cannot be ignored. For chlorhexidine, excessive or inappropriate dosages could lead to type IV and type I hypersensitivity reactions accompanied by serious anaphylaxis (Rose et al., 2019). Another concern regarding chlorhexidine is antimicrobial resistance caused by mutations in or the addition of genetic material, which indicates that chlorhexidine becomes less effective (Cieplik et al., 2019; Kampf, 2016; Zhang et al., 2019). Indinavir is associated with many adverse symptoms, including hyperbilirubinemia, renal lithiasis, and chronic renal impairment (Boubaker et al., 1998; Boyd et al., 2006; Zucker et al., 2001). It is noteworthy that a study reported that indinavir failed to inhibit the activity of the ZIKV NS2B-NS3 protease and was used as a negative control (Akaberi et al., 2020). This finding appears inconsistent with our observation of a certain level of antiviral efficacy for indinavir in both cellular and animal models. This discrepancy may stem from the following reasons. (a) Different antiviral mechanisms: Indinavir might exert its anti-ZIKV effects by targeting other stages of the viral life cycle (e.g., viral assembly) or through host-directed mechanisms, rather than by directly inhibiting the NS2B/NS3 protease. (b) Disparities in experimental systems: Biochemical enzymatic assays differ from integrated cellular/animal models in terms of drug metabolism, cellular permeability, and the complex biological milieu. Therefore, if both are used as lead compounds against ZIKV for subsequent development, efforts must be made to reduce their systemic toxicity through structural optimization, dosage form modification, or local administration strategies, and strict monitoring of drug resistance risks.

In conclusion, both indinavir and chlorhexidine, as candidate anti-ZIKV agents, still require further extensive investigation. Subsequent research may integrate methodologies such as virtual screening, antiviral activity analysis, and molecular dynamics simulations. Based on the activity requirements for ZIKV replication inhibition, this integrated approach can optimize the chemical structures of the relevant compounds and improve their safety profiles. This study may provide a new therapeutic option for ZIKV-associated diseases and offers a promising lead compound for anti-Zika virus drug development.

## Data Availability

The original contributions presented in the study are included in the article/**Supplementary Material**. Further inquiries can be directed to the corresponding authors.
