# Supplementary material for: From virtual screening to animal models: chlorhexidine and indinavir as promising anti-Zika drug candidates
Source: Front Cell Infect Microbiol. 2026 Jan 30;15:1699057. doi: 10.3389/fcimb.2025.1699057 (PMC12901475; doi:10.3389/fcimb.2025.1699057)
Supplement: Supplementary file 1 [file Table1.docx]

***Supplementary Material***

**Supplementary Figures**


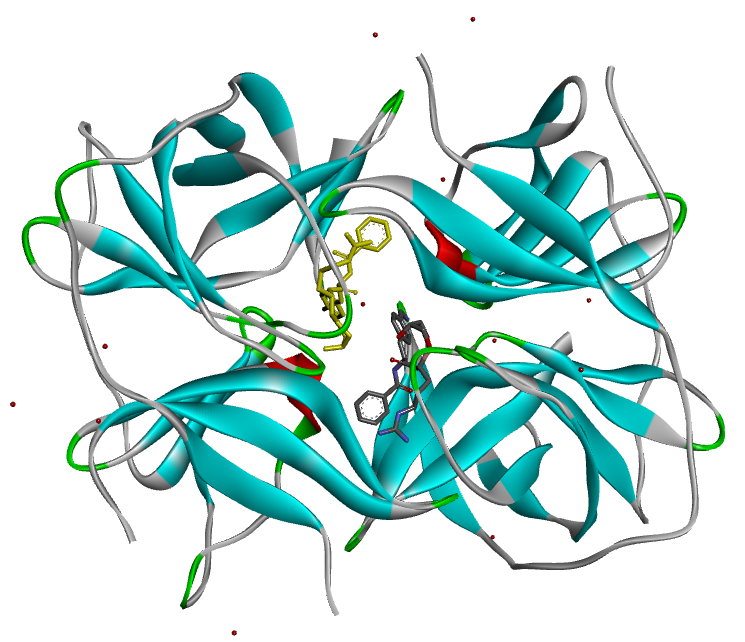


**Supplementary Figure 1.** The structure diagram of the NS2B/NS3 protein from the ZINC database. Water molecules are represented by red dots, and small molecule ligands (6T8) are represented by yellow blocks.


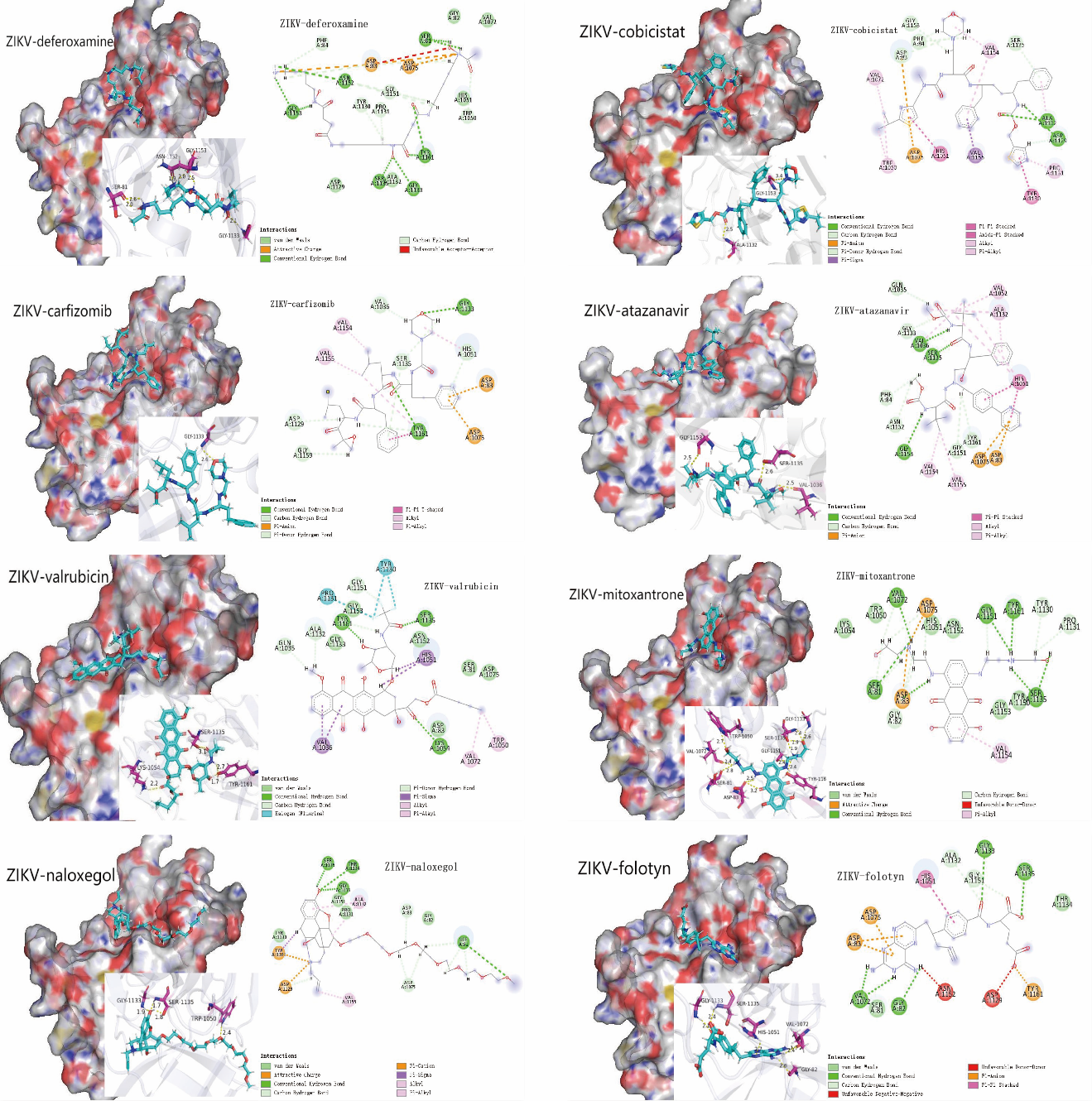


**Supplementary Figure 2**.Molecular docking results of 8 compounds.

**Supplementary Table 1**. Primers for qPCR

| Gene | Forward Primer | Reverse Primer |
| --- | --- | --- |
| ZIKV | CTATAGTCAGGCCGAGAACGC | CCCAGATTAAAGGGTGGGGA |
| GAPDH | TTCACCACCATGGAGAAGGC | GGCATGGACTGTGGTCATGA |
